# Telephone interview in urogynecology in the era of COVID-19 pandemic

**DOI:** 10.4274/jtgga.galenos.2020.2020.0131

**Published:** 2021-02-24

**Authors:** Marta Barba, Stefano Manodoro, Sara Bosio, Luca Locatelli, Matteo Frigerio

**Affiliations:** 1 University of Milano-Bicocca, Monza, Italy; 2 Department of Gynecology, ASST Santi Paolo e Carlo, San Paolo Hospital, Milano, Italy; 3 Department of Gynecology, ASST Monza, San Gerardo Hospital, Monza, Italy

**Keywords:** Telemedicine, prolapse surgery, anti-incontinence surgery, COVID-19, female pelvic medicine

## Abstract

**Objective:**

During the Coronavirus disease-2019 (COVID-19) pandemic deferable access, including non-urgent outpatient visits, have been suspended. In our practice non-urgent routine visits for pelvic floor symptom assessment were discontinued and rescheduled, and telephone interview was performed. The aim was to evaluate patients’ satisfaction for this alternative approach.

**Material and Methods:**

Telephone interviews were conducted using a validated questionnaire to investigate pelvic floor symptoms. Patients were also asked if they had other symptoms or disorders, to identify patients who may need urgent hospital evaluation. At the end of the phone call, patients were asked to score their satisfaction with the telephone interview by grading their response to three questions from 0 (minimum) to 10 (maximum). The questions were: 1) “Was the telephone interview useful to check your state of health?”; 2) “Was the telephone interview an adequate healthcare tool in consideration of COVID-19 outbreak?”; 3) “Could the telephone interview replace the conventional visit?”.

**Results:**

Fifty-three patients were evaluated. All patients showed great satisfaction with telephone interview (Q1 median: 10, IQ range: 10-10) and were similarly positive in response to the second question (Q2 median: 10, IQ range: 10-10). Although fewer patients felt that telephone interview could replace conventional clinic visits most remained positive (Q3 median: 7; IQ range: 6-8).

**Conclusion:**

This simple experience showed that phone interviews with validated questionnaires are appreciated and effective to safely perform interview of selected urogynecologic patients.

## Introduction

Italy was one of the countries most severely affected by Coronavirus disease-2019 (COVID-19) in the World. Since hospitals present a high risk of infection - through COVID-19 patients and asymptomatic health workers - deferable access, including non-urgent clinical activities, have been suspended (1). Thus there was a need to develop alternative solutions, as the demand for healthcare does not disappear, to continue providing healthcare for the population. These solutions included mobile clinics and home-based care, thus reducing access to hospitals, exposure to gatherings of people and consumption of protective equipment (2). One solution is telephone interview that allows patients to remain in their own home. This can be suitable for the health-care of urological patients, as previous reports have shown it to be feasible and associated with good patients satisfaction (3). In female pelvic reconstructive surgery, telephone interview has been proposed for interview of patients undergoing midurethral tapes, as a screening tool to identify patients who need conventional clinical consultation (4,5). Recently, the Phone Study compared telephonic and clinic interview results after pelvic organ prolapse and anti-incontinence surgery in female patients and demonstrated the feasibility and reliability of a telemedical procedure (6). In the era of the COVID-19 pandemic, telephone interview may be even more appropriate, to limit the contagion. Therefore, we decided to perform telemedicine using a symptom related questionnaire for patients with non-urgent outpatient visits suspended due to COVID-19. We hypothesized that patients would appreciate this alternative approach to maintain care continuity. Specifically, we aimed to evaluate patients’ satisfaction for telephone interview in terms of appropriateness and quality of healthcare service provided.

## Material and Methods

This study was conducted in two university hospitals in Lombardy, the Italian region worst affected by COVID-19. Patients whose pelvic organ prolapse or anti incontinence postoperative clinical consultation was suspended due to COVID-19 lockdown, scheduled from March 16, 2020 to April 30, 2020, were involved. Interview visits were rescheduled, and in the meanwhile a telephone interview was performed. Telephone interviews were conducted through a modified version of the questionnaire validated by Balzarro et al. (6), investigating prolapse symptoms, urinary incontinence, sexual dysfunction, voiding difficulties, lower urinary tract symptoms and bowel dysfunction (Table 1). Patients were also asked if they had other symptoms or disorders, to identify patients who may need urgent hospital evaluation. At the end of the phone call, patients were asked to score their satisfaction with the telephone interview on a scale of 0 (minimum) to 10 (maximum). The patients were asked only three questions, which were: 1) “Was the telephone interview useful to check your state of health?”; 2) “Was the telephone interview an adequate healthcare tool in consideration of COVID-19 outbreak?”; 3) “Could the telephone interview replace the conventional visit?”.

### Statistical analysis

Data obtained during the telephone interview assessment were statistically analyzed with JMP 9.0 (SAS, Cary, NC, USA). Continuous data are presented as median and interquantile range for non-parametric variables and as mean ± standard deviation for parametric variables, while non-continuous data as number (percentage). This study was considered exempt from local ethical committee approval as it only involved standard clinical practices.

## Results

**Figure 1:**
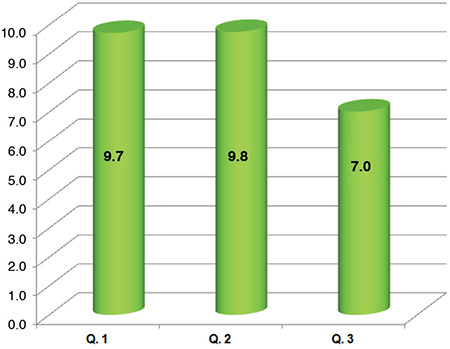
Mean satisfaction score with the telephone interview in a range from 0 (minimum) to 10 (maximum) with the following three questions: Q1) “was the telephone interview useful to check your state of health?”; Q2) “was the telephone interview an adequate healthcare tool in consideration of COVID-19 outbreak?”; Q3) “could the telephone interview replace the conventional visit?” COVID-19: Coronavirus disease-2019

In total 53 patients answered telephone calls. Mean patient age was 65.6±9.3 years. Surgical technique performed and duration of interview was very variable since it involved the activity of two different Institutions. In the cohort, prolapse repair surgery represented the main indication for interview (79.2%). All patients showed great satisfaction with telephone interview. Median scores for each question were: Q1 10; Q2 10; and Q3 7 (Figure 1). We also collected positive feedback about the supporting and reassuring effect of our calls. None of the patients described symptoms that required an urgent conventional evaluation.

## Discussion

This study evaluated the feasibility of telephone interview for patients in which non-urgent, conventional, post-operative check-up was suspended due to COVID-19. All patients showed great satisfaction with telephone interview and considered telephone interviews as an adequate tool for replacement of routine hospital visits during COVID-19 lockdown. Moreover, most were positive about the possibility of telephone interview replacing conventional check-up. We also collected positive feedback about the supporting and reassuring effect of our calls.

The COVID-19 pandemic has significantly affected the way providers care for patients, and urogynecology is no exception. Despite no clear guidelines on the use of telemedicine in female pelvic medicine and reconstructive surgery, recently an effort was made to provide guidance regarding management of common outpatient urogynecology scenarios during the pandemic (7). While surgeons usually feel compelled to check postoperative patients, there is growing evidence that patient stratification based on perioperative risk and postoperative risk may decrease the total number of conventional visits to enhance physical distancing (6). Recently, a pre-COVID-19 pandemic randomized clinical trial showed that telephone interview after pelvic floor surgery resulted in non-inferior patient satisfaction, without differences in clinical outcomes or adverse events (8).

This study confirmed that telemedical interview may allow healthcare continuity and may be particularly appropriate in the era of the COVID-19 pandemic. Phone interviews using a validated questionnaire appeared to be appreciated and an effective tool to safely perform interview of patients with functional disorders. This approach involves several advantages. The first is the “forward triage”, the capability to sort patients with urgent need for care from those who can safely postpone the conventional clinical examination. In this way it was possible to reduce community exposure for people with functional urologic disorders, which may potentially be looking for alternative health providers, such as inappropriate emergency room access or general practitioners consultation. This is extremely important, since these patients are often elderly and frail, and more prone to develop severe and life-threatening complications in case of COVID-19. Moreover, for functional disorders, telephone interview can often be adequate to address symptoms referred by patients, and set up an initial diagnostic or therapeutic path. At the same time, this procedure, acting as a screening tool, avoids neglecting serious conditions that require medical evaluation. One more aspect of the telephone interview is the capability to provide additional social support for patients living through the pandemic, who are lonely, anxious and fearful of abandonment. It was evident from our cohort that these emotions are widespread among patients compelled to stay home for days or weeks due to COVID-19 lockdown. The very high levels of satisfaction expressed by our patients can probably be explained in part by the human aspect of the conversation, besides clinical contents, that was particularly appreciated. Moreover, this telephonic approach reduced the consumption of protective equipment and clinicians’ exposure to contagion. This is particularly relevant considering that large numbers of health care workers having to quarantine would impact the capacity of health institutions to face the current COVID-19 emergency. Lastly, this telehealth approach could be conducted by quarantined health workers, with both patient and clinician at home, greatly optimizing resources and permitting uninterrupted care of established patients (9).

## Conclusion

Although this is a relatively small-scale pilot experience, we believe that the concept of telephone interview using validated clinical tools, such as checklists and questionnaires, could be potentially widespread to manage the arising limitations during the COVID-19 pandemic successfully. This is an efficient and low-resource solution that guarantees vulnerable patients’ care while protecting both patients and health providers.

## Figures and Tables

**Table 1:**
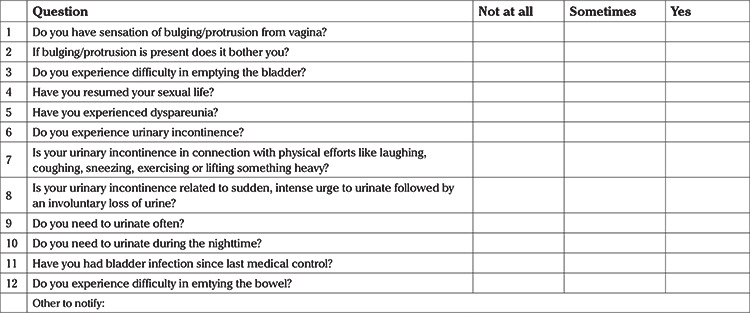
Question checklist
